# Critical real‐world patient data observation of repurposable drugs from Alzheimer's disease drug development pipeline using Target Trial Emulation

**DOI:** 10.1002/alz70859_105905

**Published:** 2025-12-25

**Authors:** Feixiong Cheng, Yadi Zhou, Yuan Hou, Jeffrey L. Cummings

**Affiliations:** ^1^ Cleveland Clinic Genome Center, Cleveland, OH USA; ^2^ Cleveland Clinic, Cleveland, OH USA; ^3^ Chambers‐Grundy Center for Transformative Neuroscience, Kirk Kerkorian School of Medicine, University of Nevada, Las Vegas, NV USA; ^4^ Chambers‐Grundy Center for Transformative Neuroscience, Department of Brain Health, School of Integrated Health Sciences, University of Nevada Las Vegas, Las Vegas, NV USA; ^5^ Kirk Kerkorian School of Medicine, University of Nevada Las Vegas, Las Vegas, NV USA; ^6^ Chambers‐Grundy Center for Transformative Neuroscience, Las Vegas, NV USA; ^7^ University of Nevada, Las Vegas, NV USA

## Abstract

**Background:**

Repurposing FDA‐approved drugs offers a promising strategy to accelerate the discovery of Alzheimer’s disease (AD) treatments. Numerous trials rely on fragmented evidence from observational studies and experiments suggesting potential benefits for AD. To address this, we used a large‐scale U.S. insurance claims database and applied target trial emulation methods to comprehensively evaluate whether repurposable drugs from **AD drug development pipeline** are associated with reduced incidence of AD or progression.

**Method:**

We conducted a population‐based cohort study using the MarketScan database (2011–2020). Two cohorts were established: (1) the MCI cohort (N=316,364), consisting of individuals with mild cognitive impairment (MCI) to study AD progression, and (2) the Over‐70 cohort (N=2,388,953), including individuals aged ≥70 without a prior AD diagnosis to examine AD incidence. FDA‐approved drugs previously tested in AD trials were identified in the Alzheimer's disease drug development pipeline derived from ClinicalTrials.gov. Trial emulation was conducted using inverse probability weighting to adjust for confounders. The outcome was AD diagnosis, evaluated through cumulative difference at 3 years and Cox‐proportional hazard model.

**Result:**

Six out of 49 drugs tested in the MCI cohort and 11 out of 55 in the Over‐70 cohort showed consistent benefits compared to drugs with the same ATC Level‐2 code (Figure). Active comparator analyses suggested potential beneficial effects of bupropion (vs. escitalopram: difference: ‐0.06 [‐0.08, ‐0.05], P=0.002; hazard ratio [HR]: 0.57 [0.49, 0.66], P<0.001), trazodone (vs. sertraline: difference: ‐0.04 [‐0.05, ‐0.02], P=0.002; HR: 0.82 [0.74,0.91], P<0.001), venlafaxine (vs. escitalopram: difference: ‐0.04 [‐0.07, ‐0.02], P=0.002, HR: 0.72 [0.62, 0.85], P<0.001), and zolpidem (vs. lorazepam: difference: ‐0.04 [‐0.06, ‐0.01], P=0.002; HR: 0.69 [0.56, 0.85], P<0.001), in reducing AD progression in the MCI cohort. Additionally, liraglutide (glucagon‐like peptide‐1 receptor agonist) significantly reduced AD incidence in the Over‐70 cohort (vs. metformin: difference: ‐0.01 [‐0.02, ‐0.003], P=0.002; HR: 0.75 [0.59,0.93], P=0.010).

**Conclusion:**

Among the repurposable drugs tested in AD trials, real‐world evidence supporting their effectiveness was limited (10%). Notably, bupropion, trazodone, venlafaxine, and zolpidem are potential candidates to reduce AD incidence. Further validation using independent patient data and clinical observations are highly warranted to establish potential clinical benefits of repurposable medicines in AD.

